# Granulocyte heterochromatin: defining the epigenome

**DOI:** 10.1186/1471-2121-6-39

**Published:** 2005-11-15

**Authors:** Donald E Olins, Ada L Olins

**Affiliations:** 1 Department of Biology, Bowdoin College, Brunswick, ME 04011, USA

## Abstract

**Background:**

Mammalian blood neutrophilic granulocytes are terminally differentiated cells, possessing extensive heterochromatin and lobulated (or ring-shaped) nuclei. Despite the extensive amount of heterochromatin, neutrophils are capable of increased gene expression, when activated by bacterial infection. Understanding the mechanisms of transcriptional repression and activation in neutrophils requires detailing the chromatin epigenetic markers, which are virtually undescribed in this cell type. Much is known about the heterochromatin epigenetic markers in other cell-types, permitting a basis for comparison with those of mature normal neutrophilic granulocytes.

**Results:**

Immunostaining and immunoblotting procedures were employed to study the presence of repressive histone modifications and HP1 proteins in normal human and mouse blood neutrophils, and in vitro differentiated granulocytes of the mouse promyelocytic (MPRO) system. A variety of repressive histone methylation markers were detectable in these granulocytes (di- and trimethylated H3K9; mono-, di- and trimethyl H3K27; di- and trimethyl H4K20). However, a paucity of HP1 proteins was noted. These granulocytes revealed negligible amounts of HP1 α and β, but exhibited detectable levels of HP1 γ. Of particular interest, mouse blood and MPRO undifferentiated cells and granulocytes revealed clear co-localization of trimethylated H3K9, trimethylated H4K20 and HP1 γ with pericentric heterochromatin.

**Conclusion:**

Mature blood neutrophils possess some epigenetic heterochromatin features that resemble those of well-studied cells, such as lymphocytes. However, the apparent paucity of HP1 proteins in neutrophils suggests that heterochromatin organization and binding to the nuclear envelope may differ in this cell-type. Future investigations should follow changes in epigenetic markers and levels of HP1 proteins during granulopoiesis and bacterial activation of neutrophils.

## Background

The epigenome of a specific tissue constitutes the total set of chromatin modifications existing above the level of DNA base sequence and mitotically inherited, conveying stability to the differentiated state. In mammalian cells, these epigenetic modifications consist primarily of DNA methylation, histone post-translational modifications and variants, and nucleosome remodeling mechanisms [1-5]. With the increased availability of reagents and techniques for defining epigenetic modifications, numerous studies have been published describing the epigenomes of various cell types. Examples of mammalian cells that have been studied include mouse resting B lymphocytes [6], and embryonic erythrocytes and fibroblasts [7].

Granulopoiesis, the terminal differentiation of blood granulocytes (primarily neutrophils or "polymorphs") occurs within the bone marrow and is well-described [8]. In humans the process takes about two weeks, starting from the myeloblast stage (ovoid nuclei with minimal heterochromatin), exhibiting one week of differentiation and mitosis, followed by one week of post-mitotic nuclear and cytoplasmic differentiation [9]. During the post-mitotic phase the non-dividing nucleus displays progressive chromatin condensation and nuclear shape changes. The normal human neutrophil nucleus has 3–4 lobes [8]; mouse neutrophils frequently possess ring-shaped nuclei [10,11]. These modulations of neutrophil nuclear shape and the considerable amount of heterochromatin located adjacent to the nuclear envelope (NE) depend upon normal amounts of the integral NE protein lamin B receptor (LBR; for a recent review on the structure of the NE, see [12]). Without sufficient levels of LBR, the neutrophil nucleus does not exhibit the normal lobulation or ring-shape and the heterochromatin undergoes clumping removed from the NE [13,14]. Other factors involved in the differentiation of neutrophil nuclear shape include NE lamin composition and microtubule integrity (for a description of our current hypothesis, see [15]).

A recent study comparing the nuclear composition of human neutrophils with a variety of myeloid leukemias [16] concluded that normal mature neutrophils exhibit a deficiency of mono-, di- and trimethylated histone H3 lysine 9 (H3K9), combined with an absence of heterochromatin protein 1 (HP1) α, β and γ; whereas myeloid leukemias possessed all of these markers. This observation is somewhat puzzling, since LBR has been shown to interact with HP1 proteins [17,18], and HP1 has been suggested to mediate the association between heterochromatin and LBR at the NE [19,20]. Furthermore, since methylated H3K9 is a well-studied repressive epigenetic modification [21] and trimethylated H3K9 is concentrated at pericentric and centric constitutive heterochromatin [22,23], the apparent absence of methylated H3K9 and HP1 would imply a unique combination of factors in the epigenome of granulocytes.

In the present investigation, we demonstrate that human and mouse granulocytes do possess methylated H3K9 (and other methylated histones), as well as low amounts of HP1 γ. The same spectrum of epigenetic markers (including detectable levels of HP1 α, β and γ) was observed in undifferentiated and granulocytic forms of retinoic acid (RA) treated mouse promyelocytic cells (MPRO) [24], which undergo complete and normal differentiation in vitro [25]. Therefore, excepting the reduced amounts of HP1 proteins, the global epigenome of granulocytes appears to be more consistent with other mammalian cell-types and various myeloid leukemias, than has been previously suggested [16]. In the present study, mouse blood and MPRO granulocytes revealed clear co-localization of trimethylated H3K9, trimethylated H4K20 and HP1 γ with pericentric heterochromatin. This observation agrees with earlier observations of co-localizated trimethylated H3K9 and trimethylated H4K20 in pericentric heterochromatin of various cultured mouse cells [23,26].

## Results

### Peripheral blood granulocytes possess various types of histone methylation

**Figure 1:**
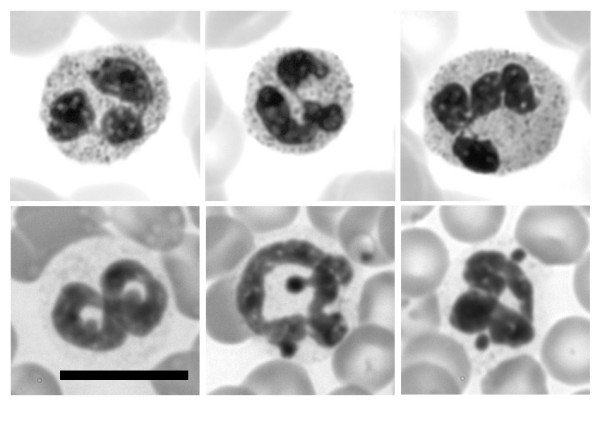
**Wright-Giemsa stained smears of human and mouse blood**. Selected neutrophils are shown (top row, human; bottom row, mouse). Note that human neutrophils exhibit nuclear lobes frequently connected by thin strands. Mouse neutrophil nuclei are usually twisted ring-shapes, frequently exhibiting several small NE nodules. Scale bars: 10 μm.

Wright-Giemsa stained smears of human or mouse peripheral blood reveal the well-described nuclear morphologies of neutrophilic granulocytes (Figure 1). Human neutrophils normally exhibit 3–4 lobes; mouse neutrophils frequently show ring-shaped nuclei with numerous NE nodules. In situ hybridization studies have clearly demonstrated that nodules in female human granulocytes (called "drumsticks") contain the inactive X chromosome [27]. However, the situation appears to be somewhat different with mouse granulocytes, since nodules are more frequent than the number of X chromosomes (unpublished observations).

**Figure 2:**
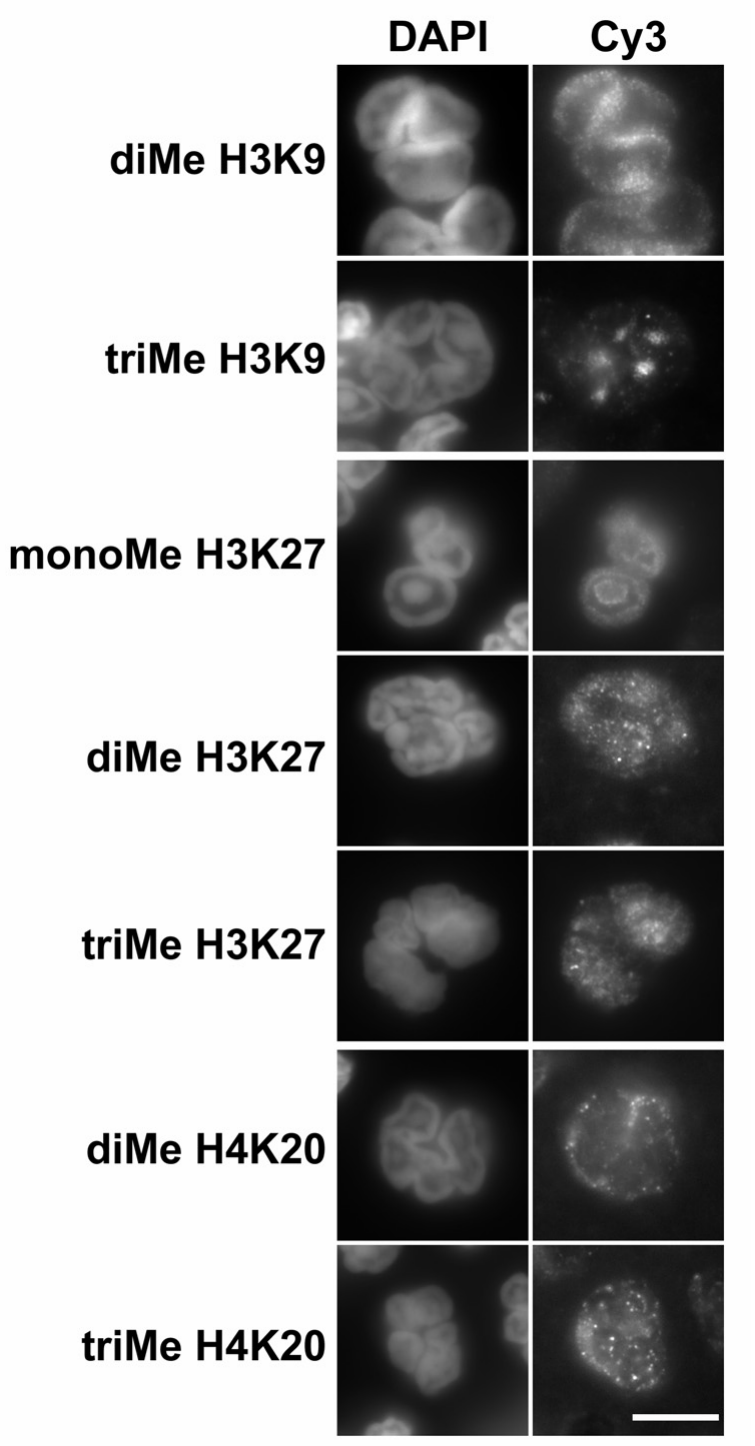
**Immunostaining of human blood neutrophils with various methylated histone antibodies**. Columns: DAPI (DNA stain); Cy3, antibody staining viewed by conventional epifluorescent microscopy. Abbreviations: monoMe, diMe and triMe, monomethylated, dimethylated and trimethylated lysine. Scale bar: 10 μm.

Slides were prepared from purified human granulocytes for immunostaining with a variety of anti-methylated histone antibodies. Some of the results from conventional epifluorescent imaging are presented in Figure 2. The purified granulocytes were centrifuged onto polylysine-coated slides, fixed with PFA, permeabilized and immunostained. All of the anti-methylated histone antibodies gave positive staining; but with varying intensities and varying patterns of localization. Anti-di- and trimethyl H3K9 and anti-mono-, di- and trimethyl H3K27 appeared to stain granulocyte nuclei. The pattern of staining by anti-trimethyl H3K9 was especially intriguing, yielding several intensely stained spots within each granulocyte nucleus. Anti-di- and trimethyl H4K20 revealed a mixture of weak nuclear and cytoplasmic staining.

**Figure 3:**
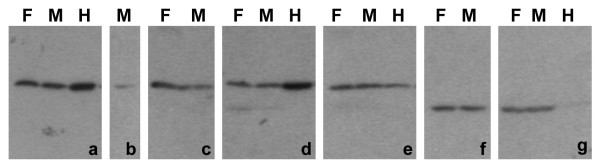
**Immunoblotting of human granulocyte extracts with various methylated histone antibodies**. Samples: F, normal female; M, normal male; H, HeLa. Panels: a, dimethyl H3K9; b, trimethyl H3K9; c, monomethyl H3K27; d, dimethyl H3K27; e, trimethyl H3K27; f, dimethyl H4K20; g, trimethyl H4K20.

Immunoblots were performed on acid extracts of purified human blood granulocytes derived from one normal male and one normal female. A gallery of representative blots, including acid extracted HeLa cells, is presented in Figure 3. Similar granulocyte protein loads were employed in all blots; Hela protein loads were also unchanged. All of the anti-methylated histone antibodies gave positive staining of varying strengths and with varying intensities relative to the HeLa extract. An indication of reaction strength was obtained by noting the exposure times of the films. Consideration of both immunostaining and immunoblotting data yielded a qualitative assessment of the antibody reactions, and is summarized in Table 1. With respect to anti-trimethyl H3K9, it is conceivable that the contrasting results, comparing immunostaining with immunoblotting, reflects high local concentrations within the pericentric heterochromatin, but low total amounts of the protein modification in the nucleus.

**Table 1:** Summmary Of Granulocyte Immunochemistry*.

**ANTIBODY**	**HUMAN BLOOD GRANULOCYTES**	**MOUSE BLOOD GRANULOCYTES**	MOUSE MPRO CELLS**
dimethyl H3K9	+ N	+ N	+ N
trimethyl H3K9	+ N g	+ N p	+ N p
monomethyl H3K27	+ N	+ N	+ N
dimethyl H3K27	+ N	+ N	+ N
trimethyl H3K27	+ N	+ N	+ N
dimethyl H4K20	+/- N, C	+/- N	+/- N
trimethyl H4K20	+ N, C	+ N p	+ N p
			
HP1 α	0^‡^	0	+/- N, C
HP1 β	0^‡^	0	+/- N, C
HP1 γ	+/- N, C	+ N p	+ N p

*The results listed represent a qualitative assessment, combining data from immunostaining and immunoblotting. In the case of normal mouse blood, due to inadequate enrichment of granulocytes, no immunoblotting was performed.

** Identical immunostaining and immunoblotting results were obtained with undifferentiated and RA differentiated MPRO cells.

Abbreviations: N, nuclear staining; N g, nuclear coarse granules; N p, nuclear pericentric heterochromatin; N, C, nuclear and cytoplasmic staining.

Reaction intensity: +, moderate to strong; +/-, weak; 0, negligible.

^‡ ^Moderate to strong staining in the cytoplasm only.

**Figure 4:**
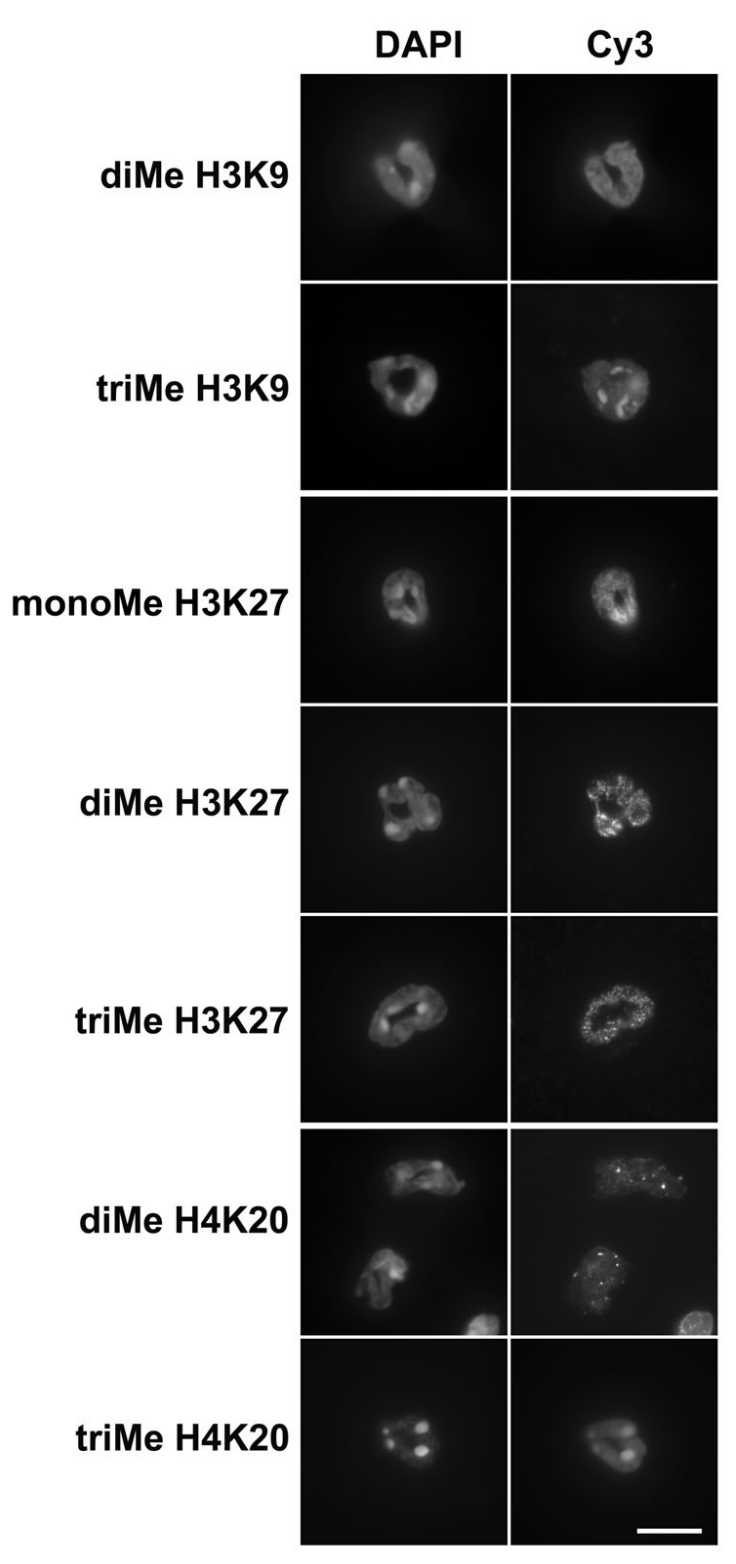
**Immunostaining of mouse blood neutrophils with methylated histone antibodies**. Columns: DAPI (DNA stain); Cy3, antibody staining viewed by conventional epifluorescent microscopy. Abbreviations: monoMe, diMe and triMe, monomethylated, dimethylated and trimethylated lysine. Scale bar: 10 μm

**Figure 5:**
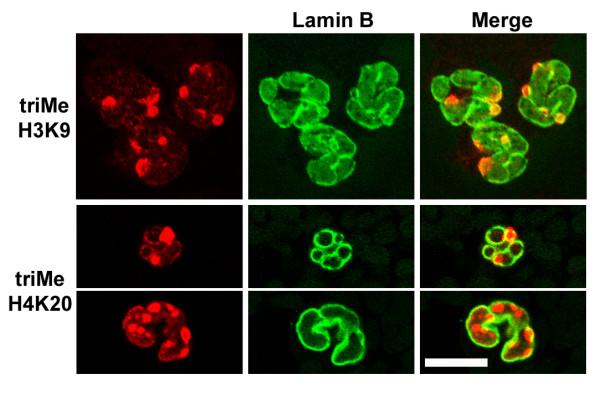
**Immunostaining of mouse blood neutrophils with methylated histone antibodies: confocal sections**. Top row: anti-trimethyl H3K9 (left); anti-lamin B (middle); merged images (right). Bottom two rows: anti-trimethyl H4K20 (left); anti-lamin B (middle); merged images (right). Slides were fixed in methanol. Scale bar: 10 μm

Heparinized whole mouse blood, diluted with DMEM medium was centrifuged onto polylysine-coated slides, fixed with PFA, permeabilized and immunostained. Granulocytes were stained with a variety of anti-methylated histone antibodies and imaged by conventional epifluorescent microscopy (Figure 4). Varying levels of reactivity with all the antibodies was noted (summarized on Table 1). Of particular interest was the clear co-localization of anti-trimethylated H3K9 and trimethylated H4K20 with DAPI-bright pericentric heterochromatin, consistent with earlier observations on mouse cells [22,23,26,28]. Confocal images of mouse blood granulocytes doubly stained for lamin B and anti-trimethylated H3K9 or trimethylated H4K20 (Figure 5) demonstrate that NE nodules appear to contain these pericentric heterochromatin markers. No immunoblots were performed on mouse granulocytes, since we were unable to obtain purified fractions suitable for extraction.

### MPRO granulocytes possess various types of histone methylation

**Figure 6:**
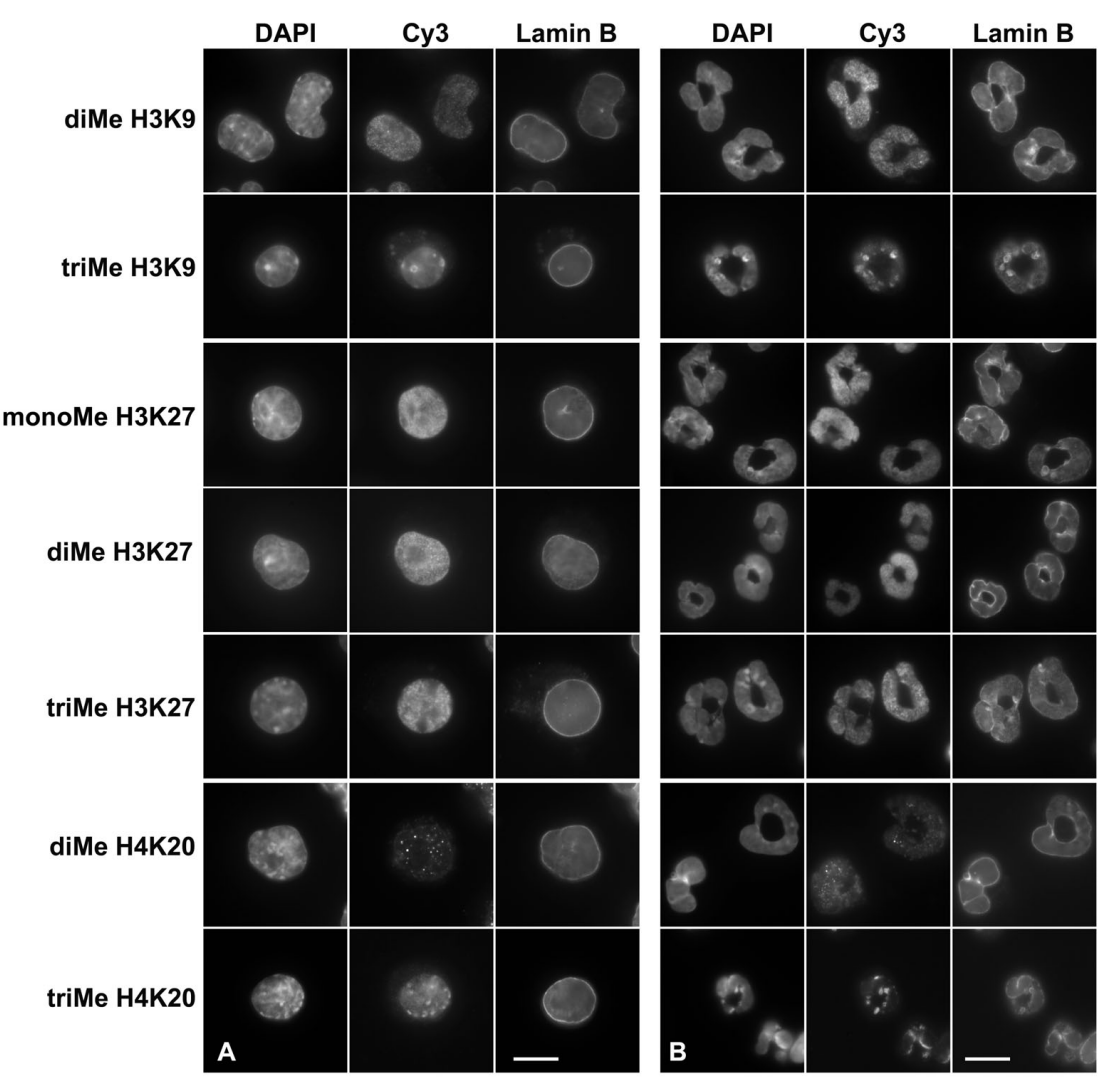
**Immunostaining of undifferentiated and granulocytic MPRO cells with various histone methylation antibodies**. Panels: A, undifferentiated cells; B, granulocyte cells (4 days of RA). Columns: DAPI (DNA stain); Cy3, anti-methylated histone staining; Lamin B, FITC staining. Rows: monoMe, diMe and triMe, monomethylated, dimethylated and trimethylated lysines. Scale bar: 10 μm.

The same panel of methylation-specific anti-histone antibodies was tested on undifferentiated and RA-differentiated MPRO cells by immunofluorescent and immunoblotting techniques. Figure 6 presents the results of immunostaining, with comparative staining by anti-lamin B and DAPI. As with mouse blood granulocytes (Figures 4 and 5), MPRO granulocytes (or undifferentiated cells) were strongly stained by anti-trimethyl H3K9 and anti-trimethyl H4K20 in bright spots that co-localized to DAPI-bright pericentric regions. More generalized nuclear staining was obtained with anti-dimethyl H3K9, anti-mono-, di- and trimethyl H3K27 and, less intensely, with anti-dimethyl H4K20.

**Figure 7:**
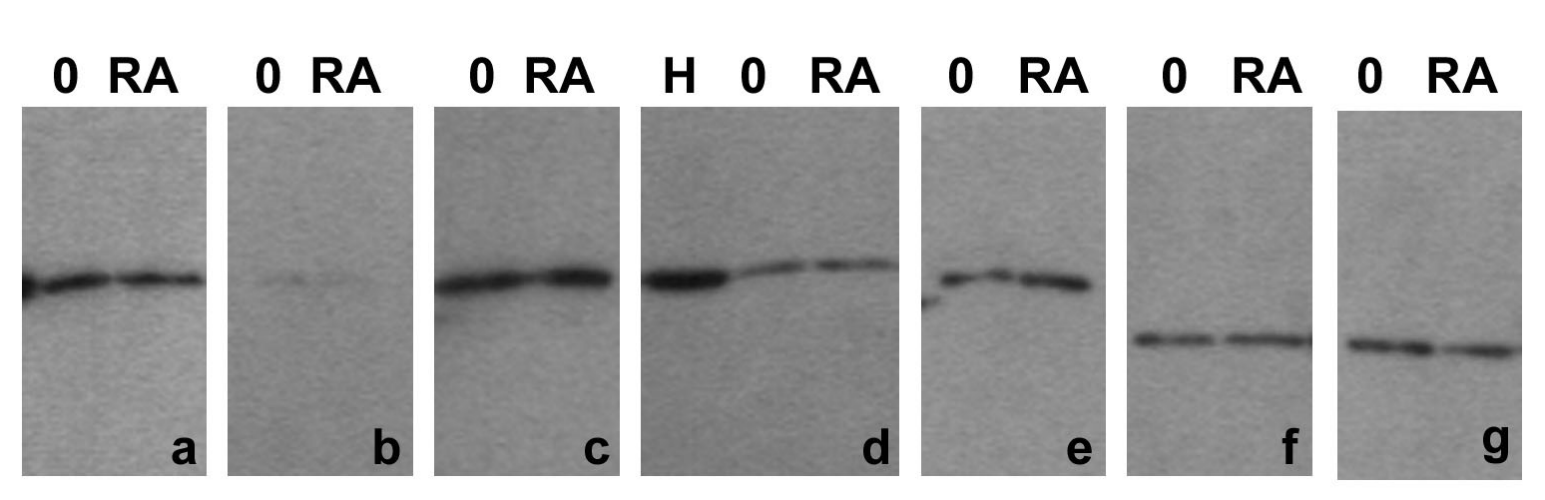
**Immunoblotting of MPRO granulocyte extracts with various histone methylation antibodies**. Samples: O, undifferentiated; RA differentiated granulocytes; H, HeLa. Panels: a, dimethyl H3K9; b, trimethyl H3K9; c, monomethyl H3K27; d, dimethyl H3K27; e, trimethyl H3K27; f, dimethyl H4K20; g, trimethyl H4K20.

Immunoblotting experiments on acid extracts of MPRO cells (undifferentiated "O", and granulocytes "RA") are presented in Figure 7. As with the previous immunoblotting data on extracts of human granulocytes (Figure 3), the same protein loads were used in all gels and the reaction intensities shown are from different exposure times. The qualitative assessment of antibody reactivity, combining immunostaining and immunoblotting data is presented in Table 1. No systematic changes in reaction intensities were observed comparing undifferentiated with RA-treated granulocytic MPRO cell extracts. Also, as described for human granulocyte extracts, anti-trimethyl H3K9 displayed very weak immunoblotting reactivity. Again we suggest that the low cellular level of trimethyl H3K9 may yield strong immunostaining because of a high local concentration in the pericentric heterochromatin.

### Peripheral blood granulocytes are deficient in HP1 proteins

**Figure 8:**
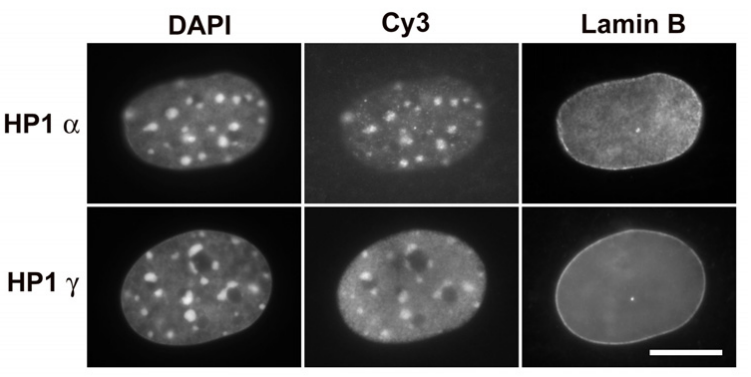
**Immunostaining of mouse NIH 3T3 cells with HP1 antibodies**. Columns: DAPI (DNA staining); Cy3, antibody staining of HP1 viewed by conventional epifluorescent microscopy; Lamin B, FITC staining. Note the clear co-localization of HP1 staining with the DAPI-bright pericentric heterochromatin. Scale bar: 10 μm

Because of difficulties that we have experienced employing commercial anti-HP1 antibodies to immunostain blood granulocytes, we decided to test the antibodies on mouse NIH 3T3 cells, where numerous authors have demonstrated localization of anti-HP1 on DAPI-bright interphase pericentric heterochromatin [7,28-30]. Results with our best antibodies for immunostaining (Chemicon anti-HP1 α and γ) of NIH 3T3 cells are presented in Figure 8. Our images closely resemble previously published data [7,30], indicating a co-localization of HP1 α and γ on pericentric heterochromatin.

**Figure 9:**
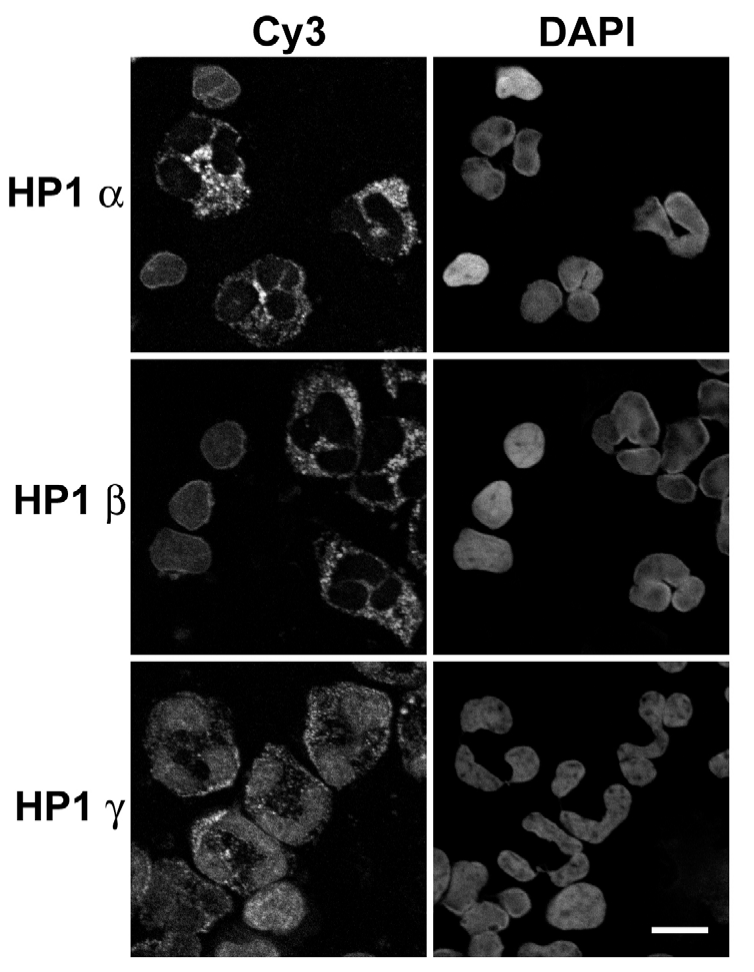
**Immunostaining of human blood buffy coat with various HP1 antibodies**. Columns: Cy3, antibody staining viewed by conventional epifluorescent microscopy; DAPI (DNA staining). Note that the monocytic nuclei exhibit staining with all monoclonal anti-HP1 antibodies (Chemicon); whereas, the granulocytic nuclei only reveal staining with anti-HP1 γ. Anti-HP1 α, β and γ appear to stain cytoplasmic granules. Scale bar: 10 μm.

Examples of HP1 α, β and γ immunostained human "buffy coat" preparations are presented in Figure 9. (Euromedex anti-HP1 antibodies were employed in this experiment.) Granulocytes of the buffy coat showed no nuclear staining by anti-HP1 α and β and only weak staining with anti-HP1 γ. Anti-HP1 α and β appeared to yield a weak reaction with granulocyte cytoplasmic granules, which was also seen using anti-HP1 γ. These granular structures do not derive from the secondary antibody (data not shown). Mononuclear cells (lymphocytes and monocytes) of the buffy coat provided an internal positive control for nuclear staining by anti-HP1 antibodies.

**Figure 10:**
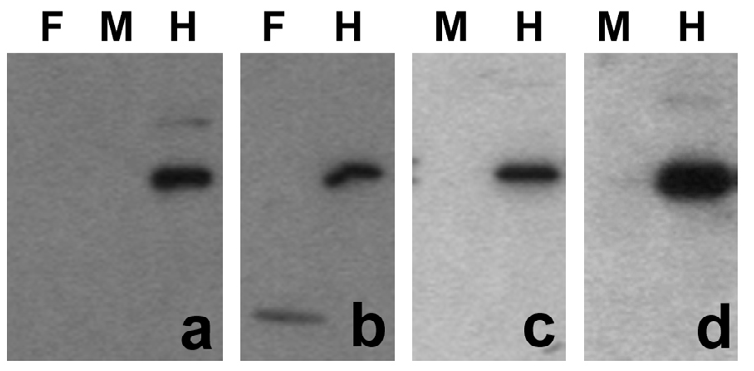
**Immunoblotting of human granulocyte extracts with various HP1 antibodies**. Samples: F, normal female; M, normal male; H, HeLa. Panels: a, monoclonal anti-HP1 α (Upstate); b, monoclonal anti-HP1 β (Chemicon); c, rabbit anti-HP1 β (Upstate); d, monoclonal anti-HP1 γ (Chemicon). Note the trace reaction of anti-HP1 γ with the granulocyte extract (panel d).

Immunoblotting experiments with whole cell extracts of human granulocytes (Figure 10) generally agreed with the immunostaining experiments; i.e., no clear evidence of HP1 α or β, an indication of trace amounts of HP1 γ. Different sources of anti-HP1 β indicated the possibility of a lower molecular weight cross-reacting antigen (the cytoplasmic granules?), which was not studied further.

**Figure 11:**
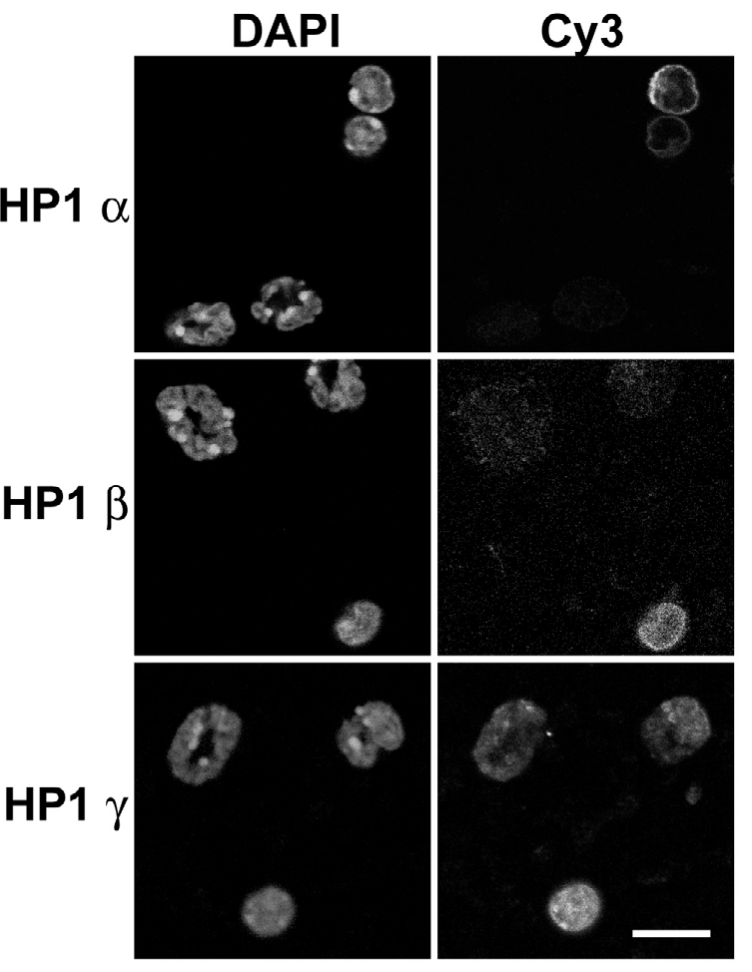
**Immunostaining of mouse blood buffy coat with various HP1 antibodies**. Columns: DAPI (DNA staining); Cy3, antibody staining viewed by conventional epifluorescent microscopy. Note that the monocytic nuclei exhibit staining with all monoclonal anti-HP1 antibodies (Chemicon). Granulocytic nuclei only reveal staining with anti-HP1 γ, which appears more concentrated at pericentric heterochromatin. Scale bar: 10 μm.

Immunostaining experiments with anti-HP1 antibodies were performed on buffy coat preparations from whole mouse blood (Figure 11). Results clearly indicated negligible staining by anti-HP1 α or β of mouse granulocytes, with clear nuclear staining of blood monocytic nuclei. However, anti-HP1 γ yielded granulocyte nuclear staining, somewhat brighter in pericentric heterochromatin; monocytic nuclei were also stained.

In summary (see Table 1), we conclude that peripheral blood granulocytes possess negligible amounts of HP1 α or β, and low levels of HP1 γ. On the other hand, blood mononuclear cells do clearly stain for all HP1 isoforms.

### MPRO granulocytes possess HP1 proteins

**Figure 12:**
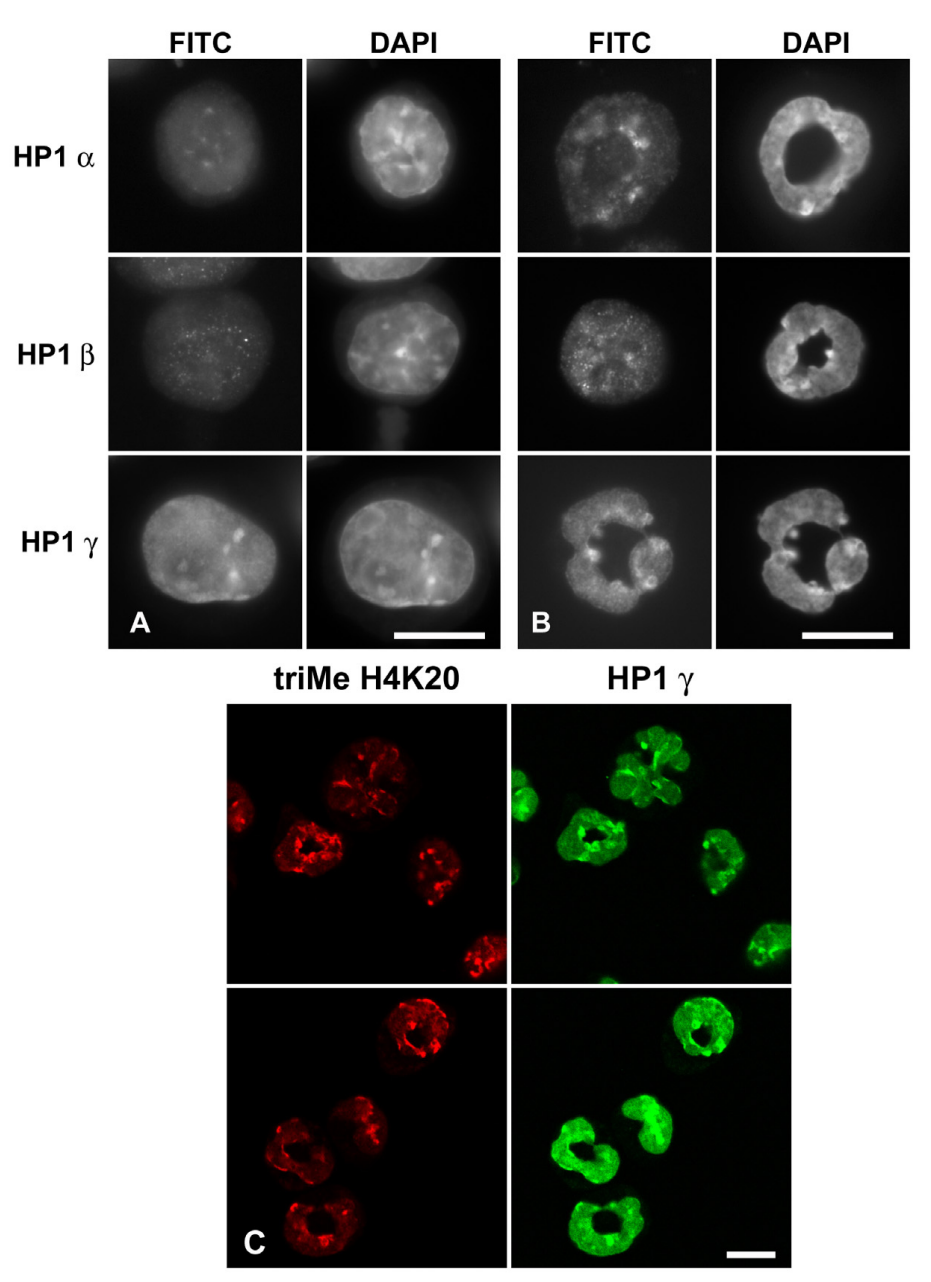
**Immunostaining of MPRO cells with various HP1 antibodies**. Panels: A, undifferentiated; B, granulocyte forms of MPRO. Columns: FITC, antibody staining viewed by conventional epifluorescent microscopy; DAPI (DNA staining). Cells were stained with anti-HP1 α, β and γ. Note the co-localization of anti-HP1 γ with DAPI-bright pericentric heterochromatin. Panel C: confocal images demonstrating co-localization of anti-trimethylated H4K20 with anti-HP1 γ stained granulocyte pericentric heterochromatin. Scale bars: 10 μm.

**Figure 13:**
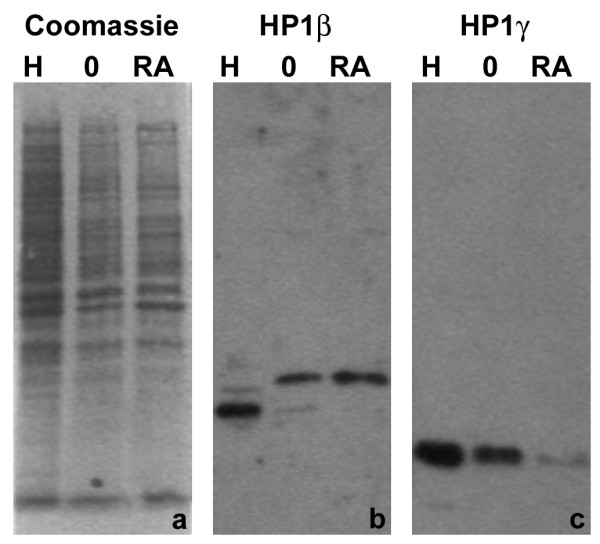
**Immunoblotting of MPRO extracts with various HP1 antibodies**. Samples: H, HeLa; O, MPRO undifferentiated cells; RA, MPRO granulocytes (differentiated 4 days). Panels: a, Coomassie blue stained membrane; b, monoclonal anti-HP1 β (Chemicon); c, monoclonal anti-HP1 γ (Chemicon). Note that MPRO HP1 β appears to be of higher mol wt, than that of HeLa cells. Also note the apparent reduction of the amount of MPRO HP1 γ during RA-induced differentiation.

Immunostaining experiments were performed on undifferentiated and granulocytic MPRO cells (Figure 12). The most convincing nuclear staining was with anti-HP1 γ, which yielded a combination of diffuse nuclear staining and focal staining, which co-localizes with DAPI-bright pericentric heterochromatin (panels A and B) and anti-trimethyl H4K20 (panel C). Both anti-HP1 α and β yielded weak staining in the nucleus and the cytoplasm. Immunoblotting experiments were conducted upon extracts of undifferentiated and RA-treated (4 days) granulocytic MPRO cells (Figure 13). An extract of Hela cells was included as a control. Convincing reactions with MPRO extracts were observed with anti-HP1 β and γ. [Anti-HP1 α (Upstate) yielded no convincing reactivity with MPRO or HeLa extracts.] MPRO HP1 β appears to possess a larger molecular weight than measured in HeLa extracts (MPRO, ~33 kD; Hela, ~28 kD) and does not appear to decrease during the differentiation process. The apparent molecular weight of HeLa and MPRO HP1 γ, as determined on 12% acrylamide SDS gels, was ~23–24 kD. Since the protein loads for the MPRO lanes was approximately the same (Figure 13, panel a), the declining reactivity comparing O and RA, suggests that the cellular level of HP1γ decreases during the granulocytic differentiation process. In summary, immunostaining and immunoblotting studies support the presence of HP1 γ in MPRO granulocyte nuclei, with weaker evidence pertaining to HP1 α and β (Table 1).

## Discussion

Blood neutrophilic granulocytes are regarded as terminally differentiated cells which are destined to undergo apoptosis, but can be activated into a short-lived migratory phagocytotic form by the presence of bacterial or yeast infections [8]. Mature neutrophils possess considerable quantities of heterochromatin [9], suggesting transcriptional repression. However, recent studies have clearly demonstrated that neutrophil activation by bacteria or bacterial lipopolysaccharide results in rapid and extensive changes in gene expression [31-33]. Our laboratory has focused upon the cellular mechanisms of nuclear shape change and heterochromatin distribution occurring during granulopoiesis [11,13-15,34,35].

The cellular mechanisms producing progressive chromatin condensation during granulopoiesis are not understood. It seems reasonable to assume that described features of constitutive and facultative heterochromatin [21,23,26,36-41] apply to the situation of condensing granulocyte chromatin. Some of these features include: 1) hypoacetylation of histone lysines; 2) methylation of specific histone lysine residues (e.g., H3K9, H3K27 and H4K20); 3) DNA methylation; 4) chromatin binding by repressive proteins, such as HP1 α, β and γ. Our present analysis of granulocyte nuclei only focuses upon two global epigenetic features, repressive histone methylation and HP1.

### Repressive epigenetic features: histone methylation and HP1

Only one published study [16] has examined global epigenetic features of granulocyte heterochromatin. This study concluded that normal human neutrophils are deficient in methylated H3K9 and HP1 α, β and γ. Based upon our present study, we agree that there is a paucity of HP1 α and β in normal human and mouse neutrophils, but we can detect low amounts of nuclear HP1 γ. Futhermore, undifferentiated and granulocytic forms of MPRO (mouse promyelocytic) cells reveal detectable levels of HP1 α, β and γ. It might be argued that MPRO granulocytes are not normal differentiated cells, but resemble leukemic cells, which do contain all isoforms of HP1 [16]. However, MPRO cells are not derived from leukemic mice and the in vitro granulocytic differentiation induced by RA is believed to yield mature granulocytes [25], with a gene expression pattern closely mimicking normal granulopoiesis [42,43].

A possible explanation for the apparent absence of HP1 α and β in normal human neutrophils is degradation during granulocyte preparation and extraction for immunoblotting. Proteolysis is a definite problem when working with granulocyte preparations, even in the presence of high quantities of protease inhibitors (unpublished observations). Whatever the reasons, our evidence indicated that low levels of HP1 γ are detectable in normal granulocytes. The evident paucity of HP1 in the nuclei of mature neutrophils suggests that models postulating an essential role for HP1 in the establishment of heterochromatin and its adherence to the NE [20] may not be applicable to these cell-types. On this issue, we are in complete agreement with the earlier published discussion [16]. We cannot rule out the possibility that HP1 proteins present early during granulopoiesis fulfil a role in the establishment of heterochromatin, which subsequently might be maintained by low amounts of HP1 γ in the mature granulocytes.

The major disagreement between our study and the previous publication [16] concerns the existence of various isoforms of methylated H3K9. We describe immunofluorescent and immunoblotting data supporting the presence of repressive methylated H3K9, H3K27 and H4K20 in normal human and mouse granulocytes and MPRO undifferentiated and granulocytic forms. The most dramatic images were obtained with trimethyl H3K9 and trimethyl H4K20 immunostained mouse blood and MPRO granulocytes. In these situations, clear co-localization with DAPI-bright pericentric heterochromatin was demonstrated. Anti-trimethyl H3K9 yielded similar intensely stained spots within human granulocyte nuclei. But the pattern of DAPI staining in human granulocytes is quite different than observed in mouse granulocyte nuclei (compare Figures 2 and 4). In human granulocyte nuclei, the DAPI-stained heterochromatin forms a thick layer under the nuclear envelope and central masses within the nuclear lobes. Several of the methylated histone antibodies (anti-dimethyl H3K9 and anti-mono-, di- and trimethyl H3K27) appeared to generally stain human granulocyte heterochromatin.

Formation of heterochromatin in the apparent absence of HP1 has been reported previously [7]. Nucleated chicken erythrocytes possess negligible amounts of HP1 α, β and γ, reduced trimethyl H3K9 and very low amounts of trimethyl H3K27. However, reasonable levels of dimethyl H3K9 were observed. In the same study, the authors demonstrated significant levels of HP1 α, β and γ and trimethyl H3K9, but reduced trimethyl H3K27 in mouse embryonic erythrocyte nuclei. More recently, in a study of resting mouse lymphocytes [6], the authors describe low levels of HP1 β, mono-, di- and trimethylated H3K9, trimethylated H3K27 and di- and trimethylated H4K20. Activation of the lymphocytes resulted in increases in the levels of all these epigenetic marks. In a variety of mouse tissue culture cells, co-localization of trimethyl H3K9 and trimethyl H4K20 on pericentric heterochromatin has been clearly demonstrated [23,26]. A model for sequential trimethylation of H3K9 and H4K20 on pericentric heterochromatin was presented [26], involving a stabilizing role for HP1 α and β binding to trimethyl H3K9 and recruiting Suv4-20h to trimethylate H4K20. It remains to be demonstrated whether HP1 γ could also serve as the postulated intermediary, in the absence of HP1 α and β.

### Histone variants and DNA methylation

An additional epigenetic feature of granulocyte nuclei, described by us in an earlier publication [35], pertains to histone H1 subtypes and phosphorylation. In a comparison of human neutrophils to leukemic HL-60 cells, we concluded that granulocytic differentiation results in dephosphorylation of subtypes H1.4, H1.5 and H1.2. Furthermore, subtype H1.3 was observed in acid extracts of normal human granulocytes, but not in those of undifferentiated or RA differentiated HL-60 cells.

There is virtually no published information on the possible role of global DNA methylation in the establishment of granulocyte heterochromatin. Given what is known from other cell types concerning CpG methylation of pericentric heterochromatin [44,45], it seems reasonable to assume a similar role for DNA methylation in granulocytic cells. However, studies on the patterns and inhibition of DNA methylation during granulocytic differentiation of HL-60 cells suggest that methylation is not essential [46,47].

### Neutrophil nuclear deformability

The present experiments, combined with earlier observations, provide a basis for understanding the mechanism of granulocyte nuclear drumstick or nodule formation observed in human or mouse blood smears. It appears that nodules often contain pericentric heterochromatin (mouse) or the inactive X chromosome (human [27]). We have previously suggested that the NE of granulocytes is highly deformable due to low levels of lamins A and B1 [11,15]. This view is based upon observations on HL-60 cells [15,34,48] and earlier studies of mouse granulocytes [49,50]. Absence of lamin A in the NE has been shown to increase NE deformability in mechanical strain tests [51], consistent with conclusions from laminopathies [12]. There is now clear and convincing evidence that the composition and integrity of the lamin polymer strongly influences the NE strength [52]. Our view is that drumsticks and nodules are artifacts of the flattening of granulocyte nuclei, reflecting the more rigid heterochromatic foci pushing against a deformable NE.

It seems reasonable to speculate that the altered composition of the granulocyte NE (i.e., paucity of lamins A and B1, and elevation of LBR [35]) is an essential part of the mechanism of nuclear lobulation [15] or formation of ring-shaped nuclei [10,11]. Both the increased deformability of the NE and nuclear lobulation (or ring-shape) may facilitate migration of activated neutrophils through blood vessel endothelia and tight tissue spaces towards a site of infection. The importance of granulocyte nuclear lobulation for chemotactic migration through narrow pores and tight spaces was demonstrated in a study of individuals with Pelger Huet anomaly [53]. We have shown NE deficiency of LBR in human Pelger Huet anomaly [13] and mouse ichthyosis [14], resulting in granulocyte nuclear hypolobulation. One additional functional consequence of increased granulocyte NE deformability may be to facilitate the formation of neutrophil extracellular traps (NETs) [54]. These structures appear to be an extruded complex of chromatin and elastase that traps and degrades invading bacteria.

## Conclusion

Mature blood neutrophils possess considerable heterochromatin containing a variety of repressive histone methylation markers: di- and trimethylated H3K9; mono-, di- and trimethyl H3K27; di- and trimethyl H4K20). In addition, normal neutrophils exhibit negligible amounts of HP1 α and β, but reveal detectable levels of HP1 γ. Clear co-localization of trimethylated H3K9, trimethylated H4K20 and HP1 γ on pericentric heterochromatin was demonstrated in normal mouse blood neutrophils and granulocytic forms of mouse MPRO cells.

## Methods

### Cells enrichment and cultivation

Human peripheral blood preparations (from one normal male and one normal female) were enriched for granulocytes and monocytes by density gradient centrifugation with HISTOPAQUE 1119 and 1077 (Sigma-Aldrich, Inc.), following procedures described by the supplier. Granulocytic and monocytic fractions were counted and examined for purity using Wright-Giemsa stain (Sigma-Aldrich, Inc., St. Louis MO). For acid extraction of histones (see below), the purified granulocytes were washed in RPMI medium and used directly. For immunostaining (see below), the granulocyte and monocyte fractions were pooled yielding a "buffy coat", which was centrifuged onto polylysine-coated slides.

Mouse blood was obtained in several ways. Separate blood pools of adult NMRI male or female mice, collected in EDTA-coated syringes, were centrifuged in HISTOPAQUE gradients. However, we routinely observed that the granulocyte and monocyte bands were cross-contaminated. Therefore, the two fractions were combined to yield a "buffy coat", which was suitable for immunostaining, but not for acid extraction of histones. In other immunostaining experiments with adult male CD1 mice, small quantities (~80–100 μl) of blood were collected in heparinized micro-hematocrit capillary tubes (Fisher Scientific, Pittsburgh PA). The contents were expelled into 1.0 ml of DMEM and centrifuged onto polylysine-coated slides.

MPRO (mouse promyelocytic) cells are a male cell line derived from normal bone marrow and grown in medium containing GM-CSF [24,25]. Following the addition of 10 μM RA, MPRO cells differentiate during four days into apparently normal granulocytes with a mixture of ring-shaped and lobulated nuclear forms [11]. Acid and SDS extracts were prepared for immunoblots during the course of RA-induced differentiation; immunostaining was performed on cells centrifuged onto polylysine-coated slides.

### Antibodies and immunostaining

Antibodies were obtained from a variety of private and commercial sources. Our experience, especially with commercial antibodies, is that they frequently do not react as described and some exhibit different reactivities when the same antibody is obtained in different lots. The most reliable anti-methylated histone antibodies were generously provided by Thomas Jenuwein (Research Institute of Molecular Pathology, University of Vienna, Vienna, Austria) and by David Allis (The Rockefeller University, New York City), and are currently available from Upstate (Charlottesville, VA). These are all rabbit antibodies, with the following names and Upstate catalogue numbers: anti-dimethyl H3K9, #07-441; anti-trimethyl H3K9, #07-442; anti-monomethyl H3K27, #07-448; anti-dimethyl H3K27, #07-452; anti-trimethyl H3K27, #07-449; anti-dimethyl H4K20, #07-031; anti-trimethyl H4K20, #07-463. Antibodies against the HP1 isoforms were purchased from both Upstate and Chemicon International (Temecula, CA) at various times and with varying success. We never obtained acceptable immunostaining with Upstate anti-HP1 α (#05-689) and (#07-346) or anti-HP1 β (#07-333), although the anti-HP1 β was useful for immunoblots. Only the Chemicon anti-HP1 antibodies provided useful immunostaining. Mouse anti-HP1 α (#MAB3584) and anti-HP1 γ (#MAB3450) yielded clear immunostaining of NIH 3T3 cells (Figure 8), but only the anti-HP1 γ was suitable for immunoblotting. Chemicon anti-HP1 β (MAB 3448, lot # 24120319) did not work for immunofluorescence or immunoblotting, although the same antibody obtained earlier (Euromedex, Mundolsheim, France; catalogue # 1MOD-1A9-AS), while visiting the German Cancer Research Center, gave clean immunofluorescent staining on NIH 3T3 cells. Goat anti-lamin B was obtained from Santa Cruz Biotechnology (Santa Cruz, CA; catalogue # sc-6216). We have purchased this antibody several times since 1997. Recently this antibody is described as anti-lamin B1. Early lots of this antibody give rim staining of human granulocyte NE; the more recent lots have not given rim staining. We suspect that the earlier lots reacted with both lamins B1 and B2, whereas the more recent lots are more specific for lamin B1. The results presented in this paper (Figures 5, 6 and 8) utilize the earlier lots.

Immunostaining procedures have been described previously [11,15,35]. In brief, cells were centrifuged onto fresh polylysine-coated slides and the cells fixed in 4% formaldehyde (PFA) in PBS for 15 min, or anhydrous methanol (-20°, 10 min). PFA-fixed cells (most experiments) were permeabilized with 0.1% Triton X-100, washed in PBS and blocked with 5% normal donkey serum in PBS. Methanol-fixed cells (Figure 5) went directly into PBS, followed by blocking. Primary antibody dilutions followed suggestions by the supplier. Secondary antibodies were obtained from Jackson ImmunoResearch Laboratory (West Grove PA).

### Cell extracts and immunoblotting

The immunoblotting procedure has been described earlier [35]. Most of the SDS-PAGE experiments were performed with 15 or 12% precast BioRad gels. Secondary HRP-conjugated antibodies were obtained from Jackson ImmunoResearch Laboratory. Several different ECL exposures were collected on X-ray film and subsequently scanned with a BioRad Chemi Doc.

## Abbreviations

RA, retinoic acid; NE, nuclear envelope; MPRO, mouse promyelocytic cells; LBR, lamin B receptor

## Authors' contributions

ALO performed the microscopy and prepared the figures. DEO performed the tissue culture, immunostaining and immunoblotting. Both authors were involved in the conception of the study and have read and approved the final manuscript.
